# Toward the Next Generation of Digitalization in Agriculture Based on Digital Twin Paradigm

**DOI:** 10.3390/s22020498

**Published:** 2022-01-10

**Authors:** Abozar Nasirahmadi, Oliver Hensel

**Affiliations:** Department of Agricultural and Biosystems Engineering, University of Kassel, 37213 Witzenhausen, Germany; agrartechnik@uni-kassel.de

**Keywords:** digital twin, digitalization, digital farming, farm management, smart farming

## Abstract

Digitalization has impacted agricultural and food production systems, and makes application of technologies and advanced data processing techniques in agricultural field possible. Digital farming aims to use available information from agricultural assets to solve several existing challenges for addressing food security, climate protection, and resource management. However, the agricultural sector is complex, dynamic, and requires sophisticated management systems. The digital approaches are expected to provide more optimization and further decision-making supports. Digital twin in agriculture is a virtual representation of a farm with great potential for enhancing productivity and efficiency while declining energy usage and losses. This review describes the state-of-the-art of digital twin concepts along with different digital technologies and techniques in agricultural contexts. It presents a general framework of digital twins in soil, irrigation, robotics, farm machineries, and food post-harvest processing in agricultural field. Data recording, modeling including artificial intelligence, big data, simulation, analysis, prediction, and communication aspects (e.g., Internet of Things, wireless technologies) of digital twin in agriculture are discussed. Digital twin systems can support farmers as a next generation of digitalization paradigm by continuous and real-time monitoring of physical world (farm) and updating the state of virtual world.

## 1. Introduction

One of the main global challenges is how to ensure food security for the world’s growing population whilst ensuring long-term sustainable development. According to the Food and Agriculture Organization, agricultural and food productions will need to grow to feed the world population, which will reach around 10 billion by 2050 [1]. Due to the increase in world population and market demand for higher product quantity and quality standards, the issue of food security, sustainability, productivity, and profitability becomes more important. Furthermore, the economic pressure on the agricultural sector, labor, environmental, and climate change issues are increasing [2,3]. Therefore, the enhancement of efficiency through effective integrated smart technologies and techniques has been widely considered in recent years.

In this context, digital agriculture (also known as smart farming or smart agriculture) tools can support the deeper understanding of interrelations within the agricultural production system and the consequent effects on the performance of farm production while balancing human health and well-being, social and environmental aspects, and sustainability associated with agricultural system [4,5,6]. Due to advances in data generation, data processing and human-computer interactions, digital farming has progressed in recent years [7]. One of the main features of digitalization in agriculture is the introduction of innovative Information and Communication Technology (ICT), Internet of Things (IoT), big data analytics and interpretation techniques, machine learning and Artificial Intelligence (AI). 

Data acquisition and analysis in digital farming by means of smart technologies are supporting complex decision-making approaches [8,9]. They enhance final productivity, reduce costs, and optimize the decision-making process. Furthermore, ICT tools present advantages for on-farm management, efficiency, quality control, and the food supply chain as well as decision support tools [10]. The AI and big data support better and precise farm monitoring, data acquisition and analytics, improve information extraction from sensors as well as farm management [11]. For instance, crop health and productivity can be monitored and controlled using advanced AI and deep learning techniques [12]. Data-driven approaches augment on-farm decision-making capabilities, improve crop yield, reduce losses, and therefore, benefit farmers. The IoT and wireless technologies enable real-time data transferring and monitoring in digital farming [13,14]. The IoT, along with cloud computing systems, can facilitate communication between software platforms and sensors, pieces of machinery, crops, and animals in digital farming. However, by increasing the number of sensors and generating large amounts of data in digital farming could cause high load on the cloud server and reduce the response speed [15]. In this context, in may be impractical to always store and process data in the cloud systems [16]. An alternative technology which has been recently introduced to the smart farming is edge-computing that enables computation at the edge of the network [17]. It helps to reduce network load and supports real-time data processing in agricultural fields. Furthermore, cyber-physical systems have been introduced through smart farming systems to develop hardware and software, improve adaptability, and safety and security of computer-based algorithms and systems [18]. It enables adaptability, practicality, security, and safety of collected information in agricultural field e.g., climate, irrigation, soil, nutrition, and yield for better management. 

According to ref. [19], digital farming approaches can provide farmers with useful information about (I) the use of fertilizers, chemicals, seeds, and irrigation management strategies, (II) the environment protection, (III) pest, climate, and crop monitoring management solutions, (IV) market demands and business conditions. However, agricultural production systems are complex, dynamic, and require sophisticated management [20]. Digitalization approaches are expected to provide more monitoring, data analysis and optimization capabilities, and further decision-making supports. 

To enhance the efficiency of these systems, an emerging paradigm has been proposed and implemented in digital agriculture, that is, digital twin. The digital twin was firstly presented by NASA for monitoring of spacecraft behavior and can be defined as a virtual or digital representation of physical systems to simulate the behavior of the physical system [21,22]. There are different definitions for digital twin available in the literature which have been reviewed by [23,24,25]. Based on the reported definitions, the component of digital twin can be characterized by physical and virtual objects, as well as a set of connections between physical and digital assets [26]. 

The physical system or physical world in agriculture is a complex and dynamic environment and includes basic information and features of the object or device such as shape, position, cooler, material, and live objects [27]. The physical system is one of the key components, and a digital twin without a physical world is a model [28], and system boundaries of a digital twin are identified based on the real physical world [29]. The physical system can be a single component of an object or the whole object with sub-components located in a physical environment [28]. The physical world in agriculture can be an animal itself or located in a farm including building, feeding strategies, number of animals [30], or a crop with different soil, climate, and irrigation conditions [22], robots and agricultural pieces of machinery, e.g., tractors, harvesters and fertilizers, as well as operators. The physical world can include a whole object (e.g., whole machine) or sub-part of the object, or a single asset of the object connected with other objects. In an agricultural context, the physical system may be some aspects of the crop, soil, and irrigation systems, or animal body. The physical world requires measurement technologies and sensors to collect and receive data from the physical object. Examples of digital twins in smart agriculture include optical sensors for plant canopy and disease [31,32], soil and weather sensors for crop [33], barn sensors such as temperature, humidity, ammonia for animals [34], Global Positioning System (GPS) and Real-Time Kinematic-Global Navigation Satellite for tracking of agricultural robots [35], and food supply chain. 

The connection between physical and virtual worlds depends on the developed digital twin. This component enables data transmission between virtual and physical systems. It interprets the collected data from the physical system and updates the state of the virtual system, and transfers feedbacks from the virtual system to the physical world [25]. The connection components can be varied depending on the source, type and volume of data, data transfer rate and speed, as well as the minimum delay between data acquisition and feedbacks. Wireless and IoT techniques have been used in digital twins of agricultural concepts to connect between physical and virtual worlds (such as [34,36,37]). 

**Figure 1:**
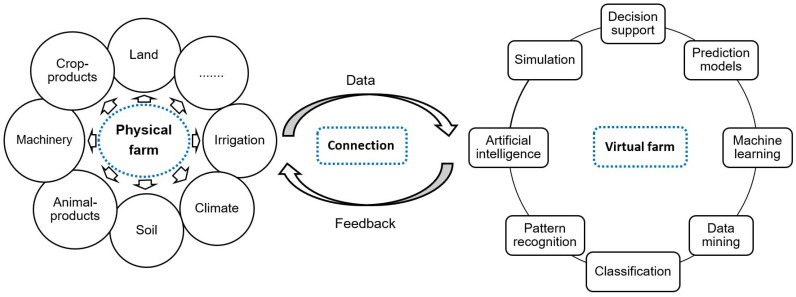
Schematic of digital twin concept for agriculture.

The models and data of the physical world are represented in a virtual system. The virtual world may also include different processing and simulation concepts, software, machine learning, data mining, and AI models. In this context, data processing and analytics by means of AI techniques to support decision-making and feedback to the physical system were suggested by some researchers [38,39]. The virtual twin may simulate and control the physical system, optimize a process, and predict unseen issues in the physical system. For example, an application layer of a digital twin reported by [22] provides real-time monitoring of weeds, crop growth, and expected yield via cloud dashboards for farmers. A schematic of the digital twin concept in agriculture is shown in Figure 1.

Although digital twin concepts in smart farming are in their infancy and early demonstration stages [22,30], there are ongoing interests in implementing this technique in the agricultural context. There are some reviews available in the literature describing digital twin concepts in the agriculture context (listed in Table 1), however, to the best of our knowledge, these works have focused on a specific part of the digital twin, and no comprehensive studies have yet been done to address the application of digital twins in soil, irrigation, agricultural farm pieces of machinery, robots, and post-harvest food processing. Therefore, this review summarizes digital twin concepts as a next-generation paradigm for digitalization in agriculture. This paper is structured in 6 sections. Section 2 illustrates the digital twin of soil and irrigation systems in smart agriculture. Section 3 covers the use of digital twin concepts for crop technologies. Section 4 illustrates digital twin concepts during post-harvest processing. Challenges and future research needs for digital twin are presented in Section 5. Finally, conclusions are discussed in Section 6.

**Table 1:** Previous review studies on digital twin in agriculture.

Concept	Sources
Agriculture-farm management	[40]
Smart farming—Hydroponics	[41]
Food processing	[42]
Food losses—supply chain of fresh products	[43]
Agri-food—societal and ethical aspects	[44]
Food processing—fresh horticulture supply chain	[45]
Agri-food supply chain	[46]
Smart farming—definition and concept	[22]
Agriculture—general application and adoption	[47]

## 2. Digital Twin in Soil and Irrigation

Monitoring and evaluation of soil quality to sustain plant productivity is the basis of land-use strategies in agricultural farms [48]. Crop health and productivity depends on the quality and properties of the soil. More detailed information about the agricultural soil may reduce the potential use of chemical fertilizer and pesticide dosages, therefore improving the underground water, protecting the environment and human health. It also supports defining plant density in a more efficient way. Digital technologies are supporting scientists to better understand and study soil in agriculture. Soil monitoring sensors such as moisture, temperature, organic matter, and soil pollutant sensors are playing critical roles in digital agriculture [49]. For instance, soil moisture information can be used to assess irrigation efficiency in agricultural fields [50]. Furthermore, to support the decision-making process of smart farming, digital soil mapping is an essential paradigm that can be defined as spatial soil information based on field and laboratory investigations coupled with soil inference systems [51]. Digital soil assessment approaches have a direct impact on crop yield and performance by identifying zones that may cause low crop yield. Digital alternative methodologies for soil survey and identifying key soil characteristics could have the possibility to quantify the trend of agricultural soil conditions [52]. 

The advancement of knowledge and technology (e.g., wireless sensors, IoT, AI) in digital agriculture could lead to digital twin paradigms of soil in agriculture. The recent development of digital soil mapping techniques may support digital twins by digital representation of knowledge obtained from the soil in virtual entrainment [53]. For instance, digital soil mapping could be used to describe soil variation in digital twins using information from complex soil variation at a specific depth, time, and special locations [52]. 

Additionally, the decision about crop management depends directly on the crop water requirements, soil properties, and availability of water. In order to manage soil and crop requirements in smart farming, digital technologies have been used to meet the requirement of smart or precise water management strategies. Wireless system networks, IoT, edge-computing, local weather-based controllers, and soil sensors are some of the digital tools based on smart irrigation systems. The mentioned tools can be used in the digital twin of soil and irrigation systems. For example, ref. [37] developed a digital twin concept for smart water management in the agricultural domain. Information of air and ground temperature, and humidity sensors, soil moisture, and ambient light as well as geospatial position sensors were collected. An IoT system was used to connect the cloud and the physical system. A virtual environment including decision-making tools and models was designed to inform the data collected by connection device (the IoT system) and to send feedback to the physical system. They also presented a digital twin system architecture including monitoring devices (i.e., soil probe, weather information, irrigation system, machines, and other equipment) in a physical system (farm) with could serve as a connection between the physical and virtual systems to visualize satellite and drone images. 

In another study, to evaluate and forecast plants’ irrigation requirements, and support irrigation and water distribution planning, a digital twin for a smart water management system was developed by [54]. Data of the physical world (agriculture field) such as weather, fertilizer, and soil type as well as information from developed models that simulate the behavior of soil and crops were considered as input data for the digital twin. The digital twin concept also consisted of a Soil Agent (includes hydrological models and soil data), Crop Agent (includes crop models and evaporation data), and a Field Avatar, which is a digital representation of the field such as geological models and weather data [54]. In their developed digital twin concept, the information from Soil Avatar and Crop Avatar feed into the Field Avatar, and an IoT system was used for data transformation and connection between the physical and virtual worlds. 

Due to increase in world population, water and energy management, storage, and proper distribution of water become more essential for water users in agricultural sectors, which can be managed through a collective irrigation system [55]. A digital twin of water systems coupled with big data can reduce risk and uncertainty of water management, explore consumption patterns, and optimize operation planning [56]. Furthermore, in a collective irrigation system, improvement of water efficiency could help to reduce water losses. In this context, a digital twin concept was created using field and laboratory tests of a collective irrigation system network to evaluate energy, pumping facilities, water losses and water use efficiencies [57]. The developed digital twin methodology was based on information from the physical system, i.e., infrastructure data, acquired information through telemetry, data analytics from laboratory tests and field measurements, IoT data transferring as connection, energy balance, water balance, and hydraulic model in the virtual system. It was found that the digital twin of the irrigation management system made it possible to understand system processes, maintenance, and management strategies [57].

A digital twin of soil and irrigation systems in smart farming enables digital representation of information from agricultural soil, and provides prediction and fundamental understanding of water requirement and soil components for crop farming. Exchanging information from the soil as a physical system to a virtual system using IoT, cloud, fog, and edge-computing technologies in digital twin may allow evaluating the state of soil and irrigation systems. In particular, the edge-computing technique that saves and performs the data processing near the soil monitoring and irrigation devices can improve the performance and overcome issues of cloud-based system in digital twin concepts. Furthermore, it could offer different irrigation recommendations based on crop requirements which are not solved yet by the researchers.

## 3. Digital Twin in Crop Production

**Figure 2:**
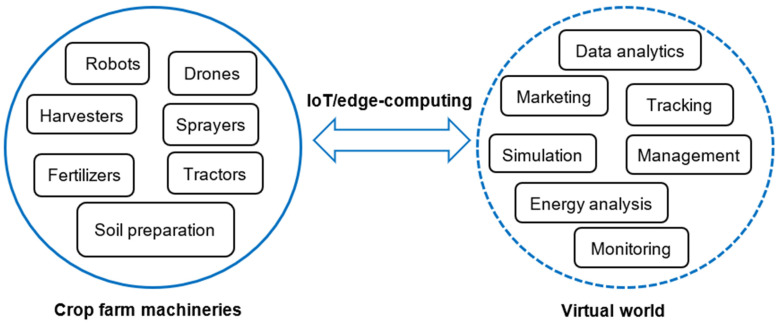
An architecture of the digital twin concept for crop production technology.

The use of digital and ICT tools in crop production technologies, in particular agricultural machineries, e.g., tractors, combine harvesters, fertilizers, and sprayers, plays an important role in the improvement of overall efficiency by reducing the cost of fuel, fertilizers, human labor, and parameters which affect production efficiency and sustainability [58]. Digitalization has modernized agricultural machinery application and management policies using collected information and advanced data analytics approaches. It allows to optimize the performance and enhance the use of advanced tools in manufacturing. For instance, based on the European Agricultural Machinery Association, a digital farm machine should be able to assist and support drivers by sending and receiving data via sensors and ICT tools, enable the best and optimal use of machinery, and the technology should facilitate the automated operation of the devices [59]. The application of AI, big data analytics, and wireless and IoT technologies have led to significant changes in farm technology roles towards the development of autonomous systems. The role of agricultural machinery in the implementation of digital agriculture was stated by [58] as data collected from sensors mounted on typical and autonomous agricultural machinery and transferred via an IoT platform. Then, the information was analyzed by data analytics such as AI, fuzzy logic, and big data analysis to support farmers, consumers, and markets [58]. In this context, combining digital tools with autonomous machines and robots could help farmers to do more effective practices and improve the quality of products [60]. Nowadays, with advancements in digital technology, the real-time visualization of smart farm equipment conditions is possible through digital twin approaches [40]. It allows contact to the system (e.g., machinery and robots), simulates the condition of the system, and monitors the behavior and operation as well as the maintenance situation of the machines (Figure 2). 

Digital twin in design and manufacturing of products (e.g., farm machinery) requires (I) geometric (e.g., size, shape) and physical properties of an object, (II) in the detailed information of the product which can illustrate dynamic processing of the object, (III) integration of geometric, physical, and process information [61]. Digital twin approaches make it possible to model, design, simulate, and develop agricultural machinery that would yield more productive machines in terms of energy and power efficiencies. For instance, it was shown that overall energy consumption of machinery could be modeled in digital twin concepts, and the effect of different factors on energy consumption can also be explored there [62]. In the agricultural context, ref. [40] reported that a commercially available digital twin platform for agricultural machinery is able to track the machines in real-time, monitor the energy consumption, economic efficiency of crop management, and trajectories of tractors by considering the specific conditions of the farm. It has also been reported that using digital twins could potentially impact the training of unskilled harvester operators and lead to high macro-economic benefits [63]. 

Within the digital farming technologies, robotics, as an important technology in crop production, has played an essential role in digitalization and has been drawing more attention in recent years. To optimize the robotic application process, reduce costs, and increase the quality and efficiency of the product, the digital twin concepts can be used for virtualization of the robot environment by introducing a remote operating system [64]. By providing simulation and remote operation possibilities and modeling various interactions between robot and environment in digital twin concepts, accuracy, performance, and flexibility may enhance, and the final product cost may decline. Ref. [65] analyzed the human-robot interactive behaviors using a digital twin platform. Their developed digital twin helps to improve operational productivity and comfort. In another study, a digital twin approach was proposed to assist the remote programming of a robot [66]. The developed digital twin system consists of a robot (as a physical object), and a gaming platform (as a virtual system) which was able to observe the motion of the robot, ease programming for complex environments as well as introduce a remote operating system for communication across different platforms [66]. In the agricultural context, an approach was recommended by [35] that the development of a digital twin paradigm for agricultural robots may improve predictive emulation of the vehicles, operational scheduling, digitalization, economic, environmental, and social sustainability in agriculture. Furthermore, the digital twin paradigm makes it possible to overcome common challenges in the control of robot components in the agriculture field. In this context, a research group demonstrated the possibility of a digital twin concept for a desktop version of an agricultural robot [67] to control the motor and indoor localization capabilities of the robot. Besides, the digital twin concept was used to predict movement and monitor the safety mechanism of the robot [67]. However, their developed digital twin concept needs different kinds of calibrations to be applicable in different environmental conditions. 

In another study, to simulate complexity of the crop production process, variability of plant, soil, environment, and technologies in the agricultural field, digital twin concepts were developed [68]. Three field robots for different agricultural applications were used to develop different digital twin concepts and optimize sensor-based autonomous navigation. It is reported that the developed concepts could provide considerable information in preparing field experiments, and better evaluation for the use and positioning of sensor systems towards demonstrating and implementation of the developed robotic technologies [68]. 

Integration of the digital twin systems with technologies and management strategies in crop production can provide a new phenomenon for digitalization in agricultural field. Management strategies can be improved and optimized by providing reliable forecasts of the key parameters in digital twins [69]. The digital twin systems can not only act as a management system, but it may also be used to revolutionize agricultural farm management strategies [40]. For instance, a digital twin concept was applied in a greenhouse to discover, analyze, and extract behavior of farmers [70]. Sensor data were analyzed using deep learning techniques to establish decision-making models to replicate expert famers’ experience for transferring to young farmers. It was found that the developed digital twin module could improve control and management strategies in crop farming [70]. In this context, the use of distributed architecture in digital twin may increase efficiency and reliability of the module by proper resource handling [71]. A distributed digital twin concept was developed to handle resources over different stakeholders and platforms in agricultural landscape [72]. It consists of different components, i.e., stakeholders, applications in agriculture and farm management, sensor data, analytics and simulation tools, virtual model, IoT, and resource registry which makes interoperable and cross-scale management possible in agricultural landscape [71]. 

In addition, the use of digital twin system as a decision support system can benefit and be adopted for crop farming applications, and optimization of products and farm system performance. A digital twin model was implemented by [36] in sustainable agriculture for monitoring and control of product qualities, adjustment of environmental conditions, identification of forecasting, and decision support scenarios. In addition, a novel approach based on digital twin paradigms was developed to forecast yield, vegetation quality, and duration of plant development [33]. Consequently, the quality of crop production could be improved due to detailed analysis and control of plant growth, and the efficiency of farms could be improved due to automation of decision support processes through the developed digital twin concept. Digital twin along with forecasting models were able to provide feedback to farmers for a better decision-making scenario in a reported study by [73]. Their proposed digital twin system consists of a monitoring system to collect environmental condition data from an underground farm, as well as data analysis and modeling techniques to identify key parameters, critical trends, and forecast operational scenarios. Furthermore, digital twin was able to optimize productivity of crops in a greenhouse environment through climate control strategies and treatments related to crop management [74]. 

Information from crop production machineries (e.g., tractors, harvesters, robots) have been used in smart farming to optimize the performance and efficiency, and reduce the fuel and energy consumption. However, the digital twin concepts collect real-time data from the devices and characterize the states of the physical object continuously. This capability makes it possible to predict and prescribe solutions using the collected information from the farm machineries. Hence, big data analytics coupled with AI models are able to detect failures in the machines before or in the early stage of when breakdowns happen. In this context, the use of state-of-the-art edge-computing systems may reduce latency by the limited amount of transmitted data and provide information from the crop production machineries such as autonomous robot, harvesters, and tractors to the digital twin concepts. The digital twin paradigm in crop farming can change production productivity, farm management, and sustainability at farm level. Advanced statistical models, machine learning and data analytic approaches can provide farmers with more precise information to make better decisions that were not possible previously. Based on the past (historical) and current continuous knowledge from crop (sensors deployed at farm) and environment data, the digital twin systems provide information about future states of the farm, and offer solutions for turning the collected information into useful and actionable on-farm knowledge. 

## 4. Digital Twin in Post-Harvest Process

Post-harvest process is a stage of agricultural products after harvesting until consuming the products, which may include transportation, drying, cooling, storage, and marketing. Through digital farming approaches, the post-harvest processes could benefit from loss reduction, improvement of monitoring and optimization of food processing, storage conditions, marketing, and transportation. Digital solutions allow monitoring real-time agri-food supply chain to increase robustness and resilience of the chain [75], and lower food waste and losses. The IoT platform supports the reduction of food losses in post-harvest processing [76], and tracking of the product through the food supply chain. To achieve food security AI and big data analytics enable data processing, optimization, and management in food and crop post-harvest stages [77], also reducing waste and improving overall profitability [78]. The ICT offers solutions to monitor and control quality criteria of food and agricultural products during post-harvest processing [43]. However, different environmental conditions, processing factors, and dynamic features of agricultural product (e.g., shape, size), environmental parameters (e.g., temperature, humidity), handling, transportation, and storage of the products influence the quality of post-harvest process [79]. 

To overcome these issues and increase the efficiency of the system, digital twin approaches have been used in post-harvest processing to continuously monitor the products and update the processing stages [80]. Digital twins, as an expanding family of digital farming could strengthen agri-food systems, affect knowledge and skills of farm management [44]. Digital twin in post-harvest processes can be defined as a digital representation of harvested agricultural products based on the information collected from the products. In this context, ref. [42] reported the digital twin concept of food processing may include: (I) data collected from a physical system (food process operation) by means of sensors that measure properties and variables of products and environmental parameters, (II) an IoT platform to provide sensor communication, data storage and big data analytics, high-performance computing, and link to the digital twin assets, (III) a simulation platform that uses input data from physical system for optimization, testing and validation of models, and provides decision supports in the virtual world. In order to benefit food processing by developing digital twin models, it is important to include accurate information representing production processes of the product, e.g., equipment, labor, and to create realistic models with all existing boundaries and barriers [81]. In a study reported by [82], a digital twin of mango fruit was developed to simulate and qualify thermal and associated bio-chemical behavior of the fruit through a post-harvest supply chain. In order to develop the digital twin concept, environmental air temperature as input was considered, and the actual supply chain conditions were mimicked within mechanistic finite element models [82]. Moreover, the impact of higher air speed on storage life, cold chain length, and delivery air temperature on the fruit quality were considered in the digital twin. It was reported that the digital twin allows to monitor and predict temperature-dependent fruit quality losses, improve refrigeration and logistic processes, consequently, it can reduce food losses [82]. Furthermore, it is reported that the digital twin can help horticultural products along with the post-harvest life, and can be used to forecast the shelf-life of agricultural products through the cold chain [45]. It can support food consumers as well as food business owners for tracking of the products, logistics, and marketing decisions; however, the existing digital twin concept needs to be enhanced by considering more biochemical and physical features [45]. Ref. [83] proposed a digital twin concept food supply chain analysis. Their developed digital twin includes: (I) a network based on knowledge from, e.g., customers, suppliers, and factories, (II) some parameters, e.g., in production, transportation, warehouses, sourcing, shipment costs, and policies, (III) various operational parameters, e.g., demand, quality, target inventory, and vehicle capacity. It was found that the developed digital twin can be used for optimization, simulation, and analysis of operation and performance changes in the food supply chain [83]. 

According to [43], digital twin in post-harvest can be considered as mechanistic, statistical, and intelligent models; however, it was found that the physics-based mechanistic digital twin concepts can evaluate the quality of fresh agricultural products better than the others. Physics-based digital twins were developed on 331 cold chain shipments of four fruits (i.e., cucumber, eggplant, strawberry, raspberry) by [84]. Based on digital twin concepts, it was found that the quality of fruits may be affected (around 43–85%) before being delivered to stores.

The post-harvest processing has improved through the application of digital solutions over the last several years. However, the use of the digital twin paradigm is receiving more attention in post-harvest food processing due to the future product quality prediction and cost reduction. The digital twin of post-harvest processes may be developed to model, optimize, represent, and characterize the design and operational parameters such as quality, safety, ingredients, shelf-life, and product status, which need to be considered by researchers in future studies.

## 5. Challenges and Future Needs

Summary of the digital twin concepts developed in the literature for different purposes in agricultural fields, including soil, irrigation, crop monitoring, robotics, farm machinery, and post-harvest processing, is presented in Table 2, Table 3 and Table 4. These tables show that the digital twin paradigm is in the early stage of research and development in the agricultural context, and future studies in terms of knowledge, technological, system development, and application aspects of digital twin concepts in different fields of agriculture should be considered.

**Table 2:** Summary of soil and irrigation digital twin concepts.

Concept	Key Components and Benefits	Source
Soil–water	Supporting precision irrigation in agriculture, better irrigation planning and water distribution, reduce crop yield losses	[54]
Soil–water	IoT-based water management platform, monitoring water pattern in soil	[37]
Water	Analyze and optimization of aquaponic systems, minimize water waste	[85]
Irrigation	Urban-integrated hydroponic system, integration of forecasting models for better decision-making assistance	[73]
Irrigation	System management and irrigation decision-making integration, water use, global energy and pumping facilities efficiency evaluation, understanding of irrigation system process	[57]
Water	Development of decision support system, enhancement of cyber-physical implementation in aquaponics	[86]

**Table 3:** Summary of the digital twin in crop production.

Concept	Key Components and Benefits	Source
Vertical farming	Environmental conditions assessment, identification of forecasting and decision support models, monitoring and optimization of agri-food lifecycle	[36]
Plant/tree	Plant condition monitoring including structure, health, stress, and quality of fruit	[31]
Robot	Analysis and performance evaluation, robot selection, and navigation	[35]
Robot	Simulation of field environment, autonomous robot navigation	[68]
Agricultural machinery	Development and advantages of business models for potato harvesting	[59]
Agricultural landscape	Resource distribution management over different stakeholders in agriculture	[72]
Crop	Forecasting yield and duration of plant development	[33]
Agricultural machinery	Development of three-dimensional geometric models, drawings of devices, mechanisms, and the attributive data	[87]
Plant	Detection of plant diseases and nutrient efficiency	[32]
Crop/hydroponic farm	Identification of crop growth parameters such as lighting, external temperature, and ventilation systems	[73]
Crop	Optimize productivity, climate control strategies, and crop treatment management in controlled environment agriculture	[74]
Robot	Co-simulation of robot environment, prediction of robot movement, and safety monitoring	[67]

**Table 4:** Summary of digital twin for post-harvest process.

Concept	Key Components and Benefits	Source
Food supply chain	Thermophysical behavior of fruit during supply chain, storage at different airflow rate, understanding, recording, and predicting losses of temperature-based fruit quality	[82]
Beverage	Predicting possible anomalies and preventing safety issues for employees	[88]
Food	Machine learning-based models for real-time response and quality predictions, maintenance, and data collection	[80]
Food supply chain	Development of practical implementation strategies, enhancing resilience food retail, and capacity management	[83]
Food	Challenges, methodologies, and opportunities for implementation of digital twin in food processing, importance of realistic and accurate models in food processing	[81]
Food	Modeling of equipment, humans, and space for fast-food producing, management of production chain, and performance evaluation	[89]
Post-harvest	Monitoring of retail stores and detection of fruit quality lost	[84]

With rapid technological and sensor development, digital twin of the agricultural soil by considering the soil quality and properties may accommodate plant productivity, health, and yield, save water, and reduce chemical usage. Many elements of the soil, irrigation, and environmental parameters in agricultural land can be continuously monitored, analyzed, and their management strategies optimized using big data analytics, machine learning models, and decision support systems embedded in the digital twin concepts. The combination of soil and irrigation digital twin approaches to record, monitor, and analyze agricultural land changes may lead to improved performance of crop farming. For instance, simulation of soil structure along with data-driven updating models could connect farmers to the farm using the IoT technology and present, in detail, pictures of parameters that impact the soil, irrigation, and crop yield. However, few studies focus on the development of digital twin concepts of agriculture soil with higher degree of flexibility as well as considering a wider range of operation than existing simulation models. Soil sensors could constantly measure and record the dynamic condition of arable soil, e.g., water holding capacity, moisture, temperature [53]. These data, along with information from soil structure and simulation techniques, can be transferred to digital twin concepts, and constant feedback from the digital world may advise real-time responses for soil and water management as well as control systems. In recent years, there has been rapid growth in the digital farming scenarios, use of remote sensing, digital soil mapping, and development of software platforms. However, researches needed to fuse the developed techniques along with the IoT, edge-computing, AI, data analytics, and simulation techniques that could lead to development of a digital twin paradigm is in an early stage and needs to be addressed in future studies. Furthermore, researchers need to consider the practical challenges of digital twin-based systems in soil and irrigation as digital twins are multi- and interdisciplinary techniques and require systems engineering perspectives [90].

Digital twin offers real-time simulation of farm machinery and robots that can benefit optimal design of the products, interaction with the environment, energy usage, and maintenance strategies. Digital twin concepts have the possibility to predict failures in farm machinery and support decision-making scenarios in plant production. Farm owners can be able to connect to the machines through virtual world for monitoring and tracking of the devices in agricultural farms. Digital twin systems are accompanied by recording a large amount of data and exchanging information between different assets; hence compiling and analyzing these data is a challenge facing farms, particularly in some rural areas with poor internet and technological infrastructures [91]. Other alternatives, e.g., Long Range technology based on wireless sensor networks communication and edge-computing could be used to mitigate internet access problems in rural areas for the connection part of the digital twin concepts [32,92]. Future opportunities for the implementation of digital twin systems in crop farm technologies could lie in the development of standards as well as data transferring and communication strategies in this context. 

The digital twin of crop production using big data collected from crop and farm machinery as well as robots, analytical and AI models, IoT, and satellite and drone information could allow simulating crop, environmental, and farm conditions in the digital world to determine unknown and unseen issues before happening in the physical world. Agricultural objects (crops in particular) need frequent updates in data to support information analysis and decision-making processes [93] which in turn can promote sustainable farming practices and save energy usage in crop productions. In this context, greater effort should be focused in the future on characterization and development of frameworks for more effective practical digital twin paradigms. In crop farming, all information may not be recorded and tracked using digital sensors; however, combining data from different sources could improve the virtual representation of the farm operation and environment [73]. Continuous monitoring of crops in digital twin systems by simulating the dynamic farm conditions and considering the effect of management, climate, and environmental conditions on the plant growth and use of data-driven models along with sensor fusion techniques could help to identify deviations from the normal conditions of the plant, and forecast growth stages to reduce risk of environmental and management effects. In future, different digital twin concepts might be applied to copy the complex physical system of crop farming in the digital world and incorporate variable sensors, data collecting strategies, modeling, forecasting, and simulation approaches in crop farming. 

In addition, digital twin concepts can support monitoring, tracking, and analysis of food through the entire supply chain. Development of a digital copy of an agricultural product to monitor post-harvest processing could be used to optimize the process, reduce energy use, labor, and food losses based on information from different sensors and simulation models. Future studies need to be carried out to consider more environmental and post-harvest product features for the development of robust digital twins [45]. Another major challenge in the development of digital twin for post-harvest processing to minimize quality losses and improve the shelf-life of the product is considering the value chain of agricultural products from farm to fork [43], which has not been addressed yet. In post-harvest processing to reduce uncertainty in digital twins and enable the consumer to trust the output of digital twin concepts, detailed experimental and data collection approaches along with numerical modeling and validation techniques need be considered. 

## 6. Conclusions

Employing digital technology has helped agricultural farm managers to improve efficiency, yield, and reduce losses. There are different types of digital farming paradigms in the literature that could be used in digital twin concepts as the next generation of digitalization in the agricultural field. The results of this review show that the digital twin concepts in agriculture and food processing have, so far, been little exploited in research. There are several research challenges and opportunities in different stages of digital farming. Digital twin paradigms can be meaningfully utilized for soil and irrigation, crop, robots and farm machinery, and post-harvest food processing in the agricultural field. In this context, most of the studies have focused on the development of digital twins by considering some limited parameters in agricultural sectors. Deploying of state-of-the-art technologies, e.g., AI, advanced statistical and optimization models, big data analytics, and three-dimensional simulation, offer further possibilities for improvement in farm management. With real-time and continuous information about agricultural assets, virtual models can predict and address unseen issues in the fields. It may support farmers to decline the economic pressure on the agricultural sector and labor issues, and help policy makers responsible for food security and environmental protection, towards strengthening the agriculture sector. In addition, it facilitates the work of researchers exploring methods to track and monitor crop farm machinery, agricultural and post-harvest products or reduce water, chemicals, and energy usage in digital farming. Although many digital twin systems in engineering, manufacturing, and health contexts have been developed, further attempts need to be considered in the agricultural context towards the development of digital twin systems that can monitor, record, and analyze data, to predict and prescribe the best decision for digital farming management. 

## Data Availability

Not applicable.
